# MRI in predicting conversion to multiple sclerosis within 1 year

**DOI:** 10.1002/brb3.1042

**Published:** 2018-08-02

**Authors:** Ayelet Eran, Melissa García, Robair Malouf, Noam Bosak, Raz Wagner, Ester Ganelin‐Cohen, Elinor Artsy, Alla Shifrin, Ayal Rozenberg

**Affiliations:** ^1^ Neuroradiology Unit Radiology Department Rambam Health Care Campus Haifa Israel; ^2^ Department of Neurology Rambam Health Care Campus Haifa Israel; ^3^ Neuroimmunology Unit Department of Neurology Rambam Health Care Campus Haifa Israel; ^4^ Neuroimmunology Unit Schneider Children's Medical Center of Israel Petah Tikva Israel; ^5^ Sackler School of Medicine Tel Aviv University Tel Aviv Israel

**Keywords:** demyelinating disease, multiple sclerosis, neuroimaging

## Abstract

**Objectives:**

Most patients diagnosed with multiple sclerosis (MS) present with a clinically isolated syndrome (CIS). We aimed to verify previously reported imaging and clinical findings, and to identify new MRI findings that might serve as prognostic factors for a second clinical episode or a change in the MRI scan during the first year following a CIS.

**Materials and Methods:**

We identified from our medical records, 46 individuals who presented with an episode of CIS, which was followed clinically and with imaging studies. A neuroradiologist blinded to the clinical data reviewed the images and recorded the number of lesions, lesion location, and the largest longitudinal diameter of the lesion.

**Results:**

One year after the first MRI, 25 (54%) patients had progressed to MS. The clinical presentation of those who were and were not diagnosed with MS was predominantly motor or sensory deficit. Patients with lesions that were temporal, occipital, or perpendicular to the corpus callosum at the first episode were more likely to have recurrence. Individuals with a combination of more than 13 lesions, with maximal lesion length greater than 0.75 cm, and a lesion perpendicular to the corpus callosum, had a 19 times higher chance of conversion MS during the following year.

**Conclusions:**

Assessment of the number of lesions, lesion location, and maximal lesion size can predict the risk to develop another clinical episode or a new lesion/new enhancement in MRI during the year after CIS. For patients with a higher risk of recurrence, we recommend closer follow‐up.

## 1 INTRODUCTION

Most patients who are eventually diagnosed with multiple sclerosis (MS) present at first with a clinically isolated syndrome (CIS) (Brownlee & Miller, 2014; Tumani, Sapunova‐Mayer, Süssmuth, Hirt, & Brettschneider, 2009). In about 50%–70% of patients, a T2‐weighted MRI taken at this phase reveals multiple asymptomatic white matter brain lesions suggestive of demyelination (Miller, Chard, & Ciccarelli, 2012; Sailer et al., 1999; Simon et al., 2015). In about 30% of patients with an abnormal MRI scan at presentation of a CIS, a second clinical attack or a change in MRI occurs within 1 year, which confirms the diagnosis of MS. However, longitudinal studies show that up to 20% of patients do not have another clinical attack for at least 20 years (Fisniku et al., 2008; Miller et al., 2012). A recent study by Brownlee, Swanton, Altmann, Ciccarelli, and Miller (2015) showed that during long follow‐up of CIS patients, 45% transform to clinical definite MS and 23% progress in MRI scan only. This wide clinical spectrum puts the treating physician and the patients in a situation of great uncertainty at the time of presentation. Therefore, we thought to try and identify MRI findings at first presentation that will assist the physician in decision making.

According to the 2015 updated guidelines of the Association of British Neurologists (Scolding et al., 2015), initiation of disease‐modifying drugs should be considered within 12 months of a significant CIS, if MRI evidence establishes a diagnosis of MS according to the 2010 McDonald criteria (Polman et al., 2011) or predicts a high likelihood of recurrent episodes. Of note, the 2010 revised criteria of McDonald (Polman et al., 2011) and MAGNISM criteria of 2016 (Filippi et al., 2016) define that a second attack can be diagnosed if there is a new T2 lesion or if there is new enhancing lesion.

Hence, the most clinically relevant question is whether or not a second episode is likely to happen in the short‐term, specifically within 1 year from CIS.

Several studies have aimed to identify predictors of clinical outcome in CIS. Most have focused on the long‐term outcome after the first attack and have compared the number and volume of lesions to the level of disability (Brex, Ciccarelli, & O'Riordan, 2002) or have used the first MRI after presentation of optic neuritis as a predictive tool for developing MS (The Optic Neuritis Study Group, 2008). Parameters that have been identified as predictors of conversion to MS during the first year include female gender (Dobson, Ramagopalan, & Giovanonni, 2012), younger age at onset, and multifocal neurological involvement (Mowry et al., 2009). Some baseline MRI characteristics may also serve as predictors of the above, including the number of lesions, the distribution, and activity of the lesions (like those determined by the Barkhof criteria: the occurrence of a gadolinium‐enhancing lesion; and the presence of a juxtacortical, infratentorial, or periventricular lesion (Barkhof et al., 1997; Tintore et al., 2006)), baseline lesion load (the proportion of brain voxels occupied by lesions), lesion distribution, and the location of the lesions within major white matter tracts (Tintore et al., 2006). A study by Menascu, Legrada, Miron, and Achiron (2017) recently checked conversion of CIS to MS in the first year in pediatric patients and found that high lesion load and location in the parietal lobe are predictors for conversion in the first year.

This study uniquely looks at conversion to MS on the first year of follow‐up in adults at a time when decisions regarding disease‐modifying drugs and frequency of follow‐up are being made. We aimed to verify known MR parameters, check additional MR parameters, and to add a scoring system for conversion to MS. Based on MRI findings and data collected from patients treated in our center, our analysis might serve as a prognostic tool for a second clinical episode or radiologic progression within the first year after a CIS.

## 2 MATERIALS AND METHODS

### 2.1 Patients

The study was approved by the institutional review board of Rambam Health Care Campus. Informed consent was waived due to the retrospective design.

We identified 50 individuals who presented with a first episode of central nervous system (CNS) inflammation at our institution during the years 2014–2016. All had had an MRI scan of the brain and/or spine at diagnosis and at least one‐year follow‐up, by an experienced neuroimmunologist. Patients with clinical or MRI characteristics of recurrent disease at presentation were excluded. The follow‐up comprised patient visits at the neuroimmunology clinic every 3 months and at least 2 MRI scans of the brain (at 3–4 months and 10–12 months) and additional MRI of the spine during that period. Based on the clinical and imaging follow‐up, the patients were divided into two groups: those who progressed and those who did not progress to MS during the year following the first MRI. Recurrence was determined based on revised McDonalds criteria 2010 as either an additional clinical attack or an interval appearance of a new lesion or a gadolinium‐enhancing lesion on MRI. Clinical data were collected in parallel with data from patients’ medical records.

### 2.2 MRI scans

All MRI scans were performed on 1.5–3 Tesla scanners (17 patients were scanned on GE Signa 1.5T magnet using an HNS coil, 16 patients were scanned on Philips Ingenia 1.5T magnet using a Multicoil coil, and 13 patients were scanned on GE discovery 750 3T magnet using HNS coil). Imaging sequences included T2 FLAIR images in at least one plane and a T1 sequence with and without intravenous gadolinium‐based contrast injection. An experienced neuroradiologist, blinded to the final clinical diagnosis, reviewed all MRI scans at presentation and recorded the following information: the number of lesions, lesion location (we evaluated periventricular, deep and subcortical white matte location, location by hemispheric lobes, brainstem and cerebellar location, gray matter location and lesions perpendicular to the corpus callusom), largest longitudinal diameter of the lesion, and enhancement pattern. The data were then analyzed based on the patients’ clinical diagnosis, to assess differences in MRI pattern at diagnosis between the groups.

Our analysis did not use the variable of lesion enhancement. This is because our center occasionally starts steroidal therapy on the day before or on the same day as the first MRI; while such treatment does not affect the size or number of lesions, it does affect the enhancement.

### 2.3 Statistical methods

Differences between patients with and without a recurrent episode were examined by Fisher's exact tests for categorical parameters, and by *t* test or Mann–Whitney *U* test for quantitative parameters. To identify the patients who were at risk for developing a relapse or a change in the MRI during the year following the first MRI, a receiver operating characteristics (ROC) curve was constructed to describe the sensitivity and the false positive rate for the average maximal length of the lesions. *p* < 0.05 was considered significant. SPSS program version 21 was used for data analysis.

## 3 RESULTS

### 3.1 Subjects

**Figure 1:**
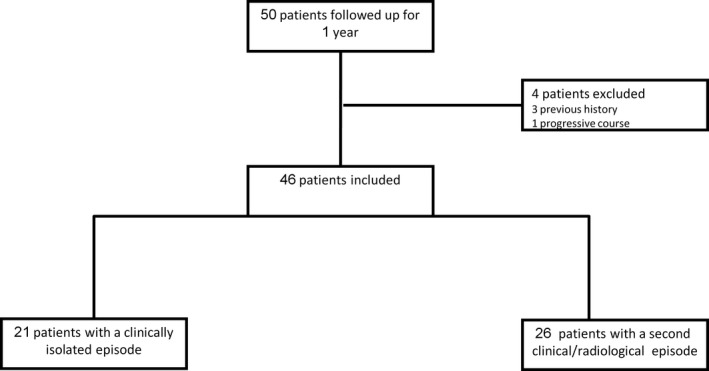
A total of 50 patients with a clinically isolated syndrome (CIS) were included in this study. Of them, 25 had a second episode, observed either clinically or via a dynamic change in MRI imaging, and 21 did not show disease progression during the first year after the CIS

We identified 50 individuals who matched the inclusion criteria. Four patients were excluded from the analysis; three exclusions were due to a thorough patient history that raised suspicion of a previous clinical episode, and the fourth was due to a progressive clinical presentation of the disease (Figure 1).

The age distribution of the 46 individuals was 19 to 42 years old. Twenty‐one had no recurrence within the first year after the isolated episode of demyelination, as determined by clinical follow‐up and by lack of a new or enhanced lesion seen via the MRI scan. Twenty‐five individuals had another clinical episode, a new lesion on MRI, or another active lesion during the year following the first episode (Figure 1). Demographic and clinical characteristics of the two groups are summarized in Table 1. The mean age was significantly younger for the patients who did than did not progress to MS: 27 versus 38 years. Women comprised 76% of the total cohort; 57% of the women, and 45% of the men had a recurrent event. Motor and sensory deficit were prominent among those who did not progress to MS; while the distribution of episodes was split between optic neuritis and motor or sensory deficit among those who progressed to MS.

**Table 1:** Demographic and clinical data of the two study groups at presentation and the clinical or radiological progression in the MS group during the first year of follow‐up

Variable	CIS	MS	*p*‐Value
Number of subjects	21	25	
Demographic characteristics			
Age (year)	38.1 ± 11.5	27.2 ± 7.7	<0.001
Female sex no. (%)	15 (71%)	20 (80%)	0.73
Medical history		1 (Diabetic)	
EDSS (expanded disability scale score) at baseline	1.881 ± 0.77	2.32 ± 0.61	0.0369
Clinical presentation type			
Optic neuritis	4	12	0.04
Transverse myelitis	5	0	0.009
Sensory/motor deficit	9	11	0.93
Other symptoms	3	3	0.82
Type of progression			
Number of patients with second clinical attack		17 (68%)	
Number of patients with dynamic in MRI		8 (32%)	
Dynamic on MRI			
Number of patients with new lesion		7 (87.5%)	
Number of patients with enhance old lesion		1 (12.5%)	
Total number of patients with enhance lesion old or new		6 (75%)	

### 3.2 Lesion location

Analysis of lesion location on MRI revealed several differences between the study groups. The parietal, temporal, and occipital lobes; the brainstem; cerebellum and the area perpendicular to the corpus callosum were all significantly more involved among patients who progressed to MS (Table 2). Three locations showed good capability of distinguishing between the groups: the temporal lobe, the occipital lobe, and the area perpendicular to the corpus callosum. Subcortical, frontal lobe, and gray matter lesions did not show the same effect.

**Table 2:** Different lesion locations between the two groups according to the first MRI scan. The numbers represent percent of patients from each group with the specific parameter

Parameter (location/length)	MS (*n* = 25)	CIS (*n* = 21)	*p*‐Value
Peri‐ventricular	100	71.4	0.006
Deep white matter	96	81	0.16
Sub‐cortical	84	61.9	0.11
Parietal lobe	100	61.9	0.001
Frontal lobe	96	76.2	0.079
Temporal lobe	88	33.3	<0.0001
Occipital lobe	84	23.8	<0.0001
Brainstem	56	19	0.016
Cerebellum	40	—	0.001
Grey matter	16	14.3	1.00
Perpendicular to corpus callosum	88	38.1	0.001
Length of the lesion	<0.75 cm	≥0.75 cm	0.009

### 3.3 Number of lesions

For patients who progressed to MS, the mean number of lesions was higher than for patients who did not progress to MS: 39.08 ± 25.39 versus 7.67 ± 6.1 (*p* = 0.0001).

### 3.4 Lesion length

**Figure 2:**
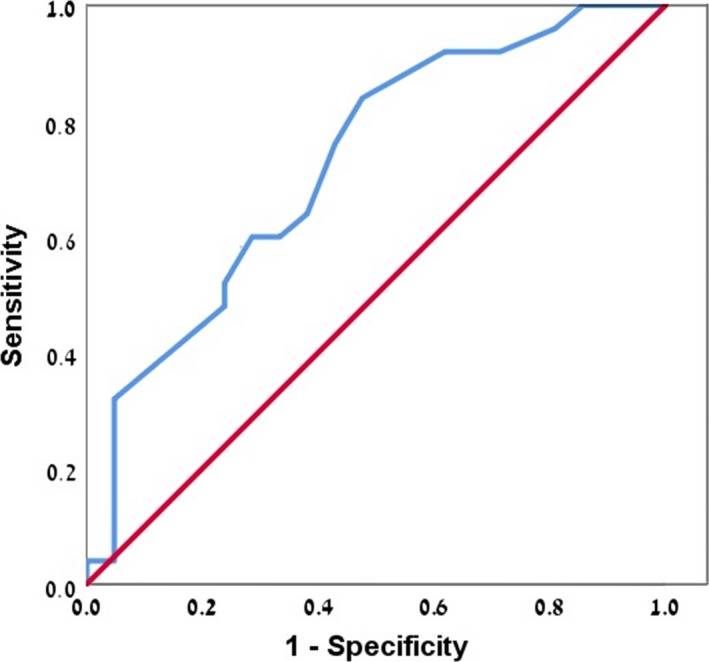
The ROC curve for the prediction of a second clinical episode or dynamic change observed on MRI imaging during the year following a first MRI, according to the size of the lesion. Higher risk was associated with a larger diameter of lesions (0.75 cm or more)

The mean maximal lesion length was greater for patients who progressed to MS: 1.42 ± 0.72 cm versus 0.94 ± 0.63 cm, *p* = 0.0218. Using ROC curves (Figure 2), we determined that a maximal lesion length longer than 0.75 cm predicted the greatest chance of dynamic clinical or radiological change during the first year.

### 3.5 Scoring

Finally, we used our findings as detailed above to create a scoring system that would predict progression to MS within 1 year following the first attack.

We found that the combination of multiple lesions (more than 13), greater lesion length (0.75 cm or more), and the presence of lesions perpendicular to the corpus callosum has a higher probability (19 times greater) to develop a second episode compared to the absence of these characteristics (*p* = 0.0001, *R* = 0.45). Our tests had a specificity of 85.7% and a sensitivity of 76%. Detailed data are presented in Table S1.

## 4 DISCUSSION

In this study, we aimed to develop an imaging tool that would enable estimating the chance of diagnosing MS during the first year after CIS, according to the first MRI scan. When an individual arrives for evaluation after a single episode of inflammation of the CNS, several decisions regarding follow‐up during the year following the event need to be made, including the frequency of MRI scans, the timing of follow‐up visits, and the decision of whether or not to start an immune‐modifying treatment. Using the parameters: number of lesions, maximal lesion length, and lesion location, we tried to identify predictors of progression to MS, as defined by the presentation of another clinical episode or a change in MRI (demonstrating a lesion in a new location or enhancement of a lesion). Consequently, for those patients with a higher chance of recurrence, we recommend considering closer follow‐up.

Studies that have examined disease progression after the first CIS, both in the short‐ and long‐term, have referred to several parameters, of which Barkhof criteria showed dominancy in baseline MRI; these were shown to be outcome predictors (Brex et al., 2002; Giorgio et al., 2013; The Optic Neuritis Study Group, 2008; Tintore et al., 2015). One study (Wottschel, Alexander, & Kwok, 2014) aimed to predict a second clinical attack after a CIS, using data collected of 74 patients and machine‐learning techniques. They illustrated that a combination of spinal cord presentation, female gender, and lesion load best predicts conversion to MS at 1 year, while other features, including age at presentation and higher lesion count, were found to be predictive only at the third year. These studies have all regarded lesion location from different perspectives (i.e., using Barkhof criteria, involvement of specific white matter tracts, and the pattern of spread). While the findings of our study are congruent with some of the previous results, they also entail novel aspects. Our research succeeded in identifying a combination of parameters, including location, the number of lesions, and the maximal length of the lesion that showed significant predictive value during the year following the first MRI. Importantly, the method is simple and can be implemented by every physician when using MRI, to identify progression to MS.

Our results can be explained in part by the aggressiveness of the disease. For example, if there are more lesions identified (greater than 13) in the first MRI scan, a higher chance of another event would be expected in the course of 1 year.

When predicting disease progression during the first year, based on lesion location, we assumed that lesions in less characteristic locations, such as the temporal or occipital lobes, would have greater correlation with aggressive disease than would those in more typical locations, such as frontal lobe or subcortical areas. However, the preferential occipital and temporal lobe distribution among patients who progressed to MS cannot be explained by the larger lobe volumes, as the frontal lobe is the largest and the occipital lobe is the smallest (Kennedy et al., 1998).

This study has a number of limitations, including small sample size and lack of perspective validation test. Another limitation is that we were unable to consider the parameter of enhancement as some of the patients in the study began steroidal therapy just before the first MRI. Otherwise, enhancement could have been included as a parameter that could potentially influence the prediction of progression. Finally, the number, location, and size of the lesions were assessed by a neuroradiologist and not by a computer program; this may have affected our results. Nonetheless, the assessment was similar for all the images.

Future studies, with a larger sample size, may further distinguish between patients with and without a clinical episode or MRI change within the year following an abnormal MRI scan. This could further improve the prognostic value of the first MRI scan and contribute to decisions regarding follow‐up frequency and possible treatment.

## CONFLICT OF INTERESTS

The authors have no conflict of interests to declare.
