# Supplementary figures and images for: MRI in predicting conversion to multiple sclerosis within 1 year
Source: Brain Behav. 2018 Aug 2;8(9):e01042. doi: 10.1002/brb3.1042 (PMC6160649; doi:10.1002/brb3.1042)

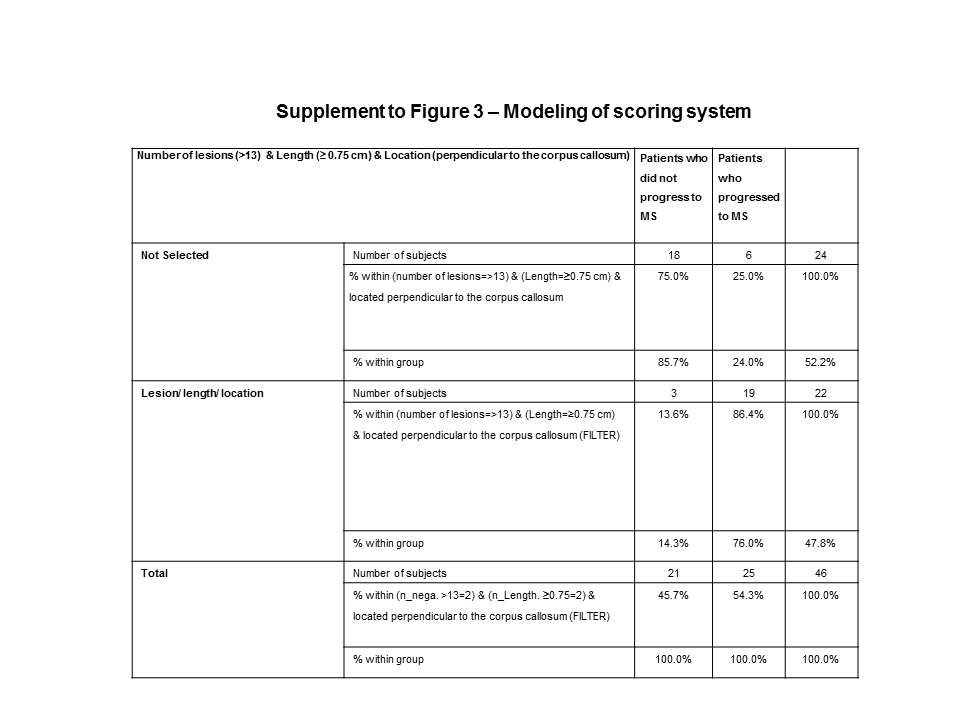

Supplement: Supplementary file 1 [file BRB3-8-e01042-s001.tif]
